# Validation of the revised diagnostic criteria for primary plasma cell leukemia by the Korean Multiple Myeloma Working Party

**DOI:** 10.1038/s41408-022-00755-w

**Published:** 2022-11-21

**Authors:** Sung-Hoon Jung, Kihyun Kim, Sang Eun Yoon, Joon Ho Moon, Dajung Kim, Hyo Jung Kim, Min Kyoung Kim, Kyoung Ha Kim, Hyun Jung Lee, Ji Hyun Lee, Sung-Hyun Kim, Kawi Han Yoo, Jae Hoon Lee, Je-Jung Lee

**Affiliations:** 1 Chonnam National University Hwasun Hospital, Chonnam National University Medical School, Hwasun, Republic of Korea; 2 Sungkyunkwan University School of Medicine, Samsung Medical Center, Seoul, Republic of Korea; 3 Kyungpook National University Hospital, School of Medicine, Kyungpook National University, Daegu, Republic of Korea; 4 Kosin University Gospel Hospital, Busan, Republic of Korea; 5 Department of Internal Medicine, Hallym University Sacred Heart Hospital, Anyang, Republic of Korea; 6 Yeungnam University Medical Center, Daegu, Republic of Korea; 7 Department of Internal Medicine, Soonchunhyang University Seoul Hospital, Seoul, Republic of Korea; 8 Kyung Hee University Hospital, Kyung Hee University College of Medicine, Seoul, Korea; 9 Department of Internal medicine, Dong-A University College of Medicine, Busan, Republic of Korea; 10 Gachon University Gil Medical Center, Incheon, Republic of Korea

**Keywords:** Myeloma, Chemotherapy

## Abstract

The International Myeloma Working Group has recently revised the diagnostic criteria for primary plasma cell leukemia (PCL) to circulating plasma cells (CPCs) ≥ 5% in a peripheral blood smear. The present study validated new criteria in patients with multiple myeloma or PCL diagnosed using the previous diagnostic criteria, who were administered immunomodulatory drugs or proteasome inhibitors as induction therapy. We analyzed the medical records of 1357 patients from eight hospitals in South Korea. The median age of the all patients was 64 years, and 187 (13.8%) had CPCs at diagnosis. Only 79 (5.8%) of the patients had ≥ 5% CPCs. The median overall survival (OS) of patients with CPCs ≥ 5% and ≥ 20% was similar, but had significantly inferior median progression-free survival (PFS) and median OS than those with CPCs < 5% (13.1 vs. 21.5 months, *P* < 0.001, and 21.5 vs. 60.9 months, *P* < 0.001, respectively). Primary PCL diagnosed using the revised criteria presented with higher total calcium levels and serum creatinine levels, lower platelet counts and frequent organomegaly and plasmacytoma at diagnosis. Univariate and multivariate analyses demonstrated that the presence of plasmacytoma and elevated serum β2-microglobulin were significantly associated with OS in primary PCL. In conclusion, the revised criterion of CPCs ≥ 5% in a peripheral blood smear is appropriate for PCL diagnosis.

## Introduction

Plasma cell leukemia (PCL) is a highly aggressive plasma cell neoplasm that are classified into primary and secondary types. Primary PCL occurs without a preceding plasma cell neoplasm at the time of diagnosis, while secondary PCL is defined as a case in which it is diagnosed during multiple myeloma (MM) treatment. PCL is rare, and is diagnosed in 0.5–4% of MM patients. Primary and secondary PCL account for 60–70%, and 30–40% of all cases, respectively [1, 2].

The diagnostic criteria of PCL proposed by Kyle [3] in 1974 required both more than 20% circulating plasma cells (CPCs) and an absolute count greater than 2 ×10^9^/l plasma cells in peripheral blood. In 2013, these diagnostic criteria were recognized by the International Myeloma Working Group (IMWG) that either criterion was sufficient for diagnosis [4]. However, these criteria were not based on prospective studies and the cut-off value of plasma cells in peripheral blood was arbitrary. Some studies have been conducted to identify the optimal diagnostic criteria of PCL, and two recent studies by Spanish group and the Mayo Clinic suggested a cut-off value of 5% [5, 6]. Based on these studies, IMWG revised the diagnostic criteria for PCL to a lower cut-off value of 5% CPCs in a peripheral blood smear (PBS) [7]. Because the revised diagnostic criteria were also based on retrospective studies, the cut-off values could require further investigation. In the present study, we investigated the proposed diagnosed criteria for PCL and evaluated the clinical characteristics and outcomes of primary PCL diagnosed using the revised diagnostic criteria.

## Patients and methods

### Patients

This study (KMMWP-2003) retrospectively evaluated the clinical and laboratory data of 1,357 patients diagnosed with MM or primary PCL using the previous diagnostic criteria at 10 hospitals in South Korea between 2001 and 2020. Patients with unconfirmed PBS at diagnosis and those who received only conventional chemotherapy for induction were excluded. The study was approved by the Institutional Review Boards of all participating institutions and was conducted in accordance with the Declaration of Helsinki.

Conventional microscopic examination of Wright-Giemsa-stained PBS was performed to evaluate CPCs. The number of CPCs in PBS before induction therapy was recorded; if multiple values were available, the highest value was used. Ambiguous results were reconfirmed by laboratory medicine specialists in each institution. The presence of plasmacytoma at diagnosis was evaluated radiologically using computed tomography, magnetic resonance imaging, or ^18^F-fluorodeoxyglucose positron emission tomography/computed tomography. Patients with plasmacytoma in an extramedullary organ or tissue were considered to have extramedullary plasmacytoma (EMD). Early death (ED) was defined as death from any cause within 4 months of diagnosis. Revised International Staging System (R-ISS) and Second Revision of the International Staging System (R2-ISS) were used to stage the disease at the time of diagnosis [8, 9]. Response to induction therapy was assessed using the IMWG uniform response criteria [10]. Patients with t(4;14), t(14;16), or del(17p) detected using fluorescence in situ hybridization (FISH) were classified as high cytogenetic risk.

### Statistical analysis

Discrete and continuous variables were analyzed using Pearson’s chi-square test and Mann–Whitney *U*–test, respectively. Progression-free survival (PFS) was calculated from the date of diagnosis to disease progression or death from any cause. Overall survival (OS) was defined as the duration between diagnosis and death from any cause or the last follow-up. Survival outcomes were evaluated using Kaplan-Meier estimates and compared using the log-rank test. Relative risk and 95% confidence intervals (CIs) were estimated using the Cox proportional hazard model. Variables with *P* values < 0.05 in univariate analyses were included in the Cox proportional hazards regression model. All statistical analyses were performed using SPSS Statistics ver. 26.0 (SPSS Inc, Chicago, IL, USA). *P* value < 0.05 was considered statistically significant for all analyses.

## Results

### Patients and treatment

The study participants had a median age of 64 years (range: 34-91); 403 (29.7%) were aged ≥ 70 years and 763 (56.2%) were male. In total, 226 (16.7%) patients were classified as R-ISS I, 59.4% as R-ISS II, and 17.5% as R-ISS III. Cytogenetic data were available for 964 patients; 16.9% were classified as high risk. At diagnosis, only 187 (13.8%) patients had CPCs; 47 (3.5%) had CPCs ≥ 20%, 3 (0.2%) had 15–19%, 9 (0.7%) had 10–14%, 20 (1.5%) had 5–9%, and 108 (8.0%) had 1–4% CPCs.

All study participants received induction therapy, while only 522 (38.5%) underwent autologous stem cell transplantation (ASCT). This study included a large number of patients aged ≥ 65 years, relatively few of whom received ASCT; 506 (73.7%) of 687 patients aged < 65 years underwent ASCT. Thalidomide- and bortezomib-based first-line regimens were administered to 430 (31.7%) and 559 (41.2%) patients, respectively. The first-line regimens included bortezomib, thalidomide, and dexamethasone (VTD) in 273 (20.1%) patients; lenalidomide and dexamethasone (Rd) in 85 (6.3%); carfilzomib, melphalan, and prednisolone (CMP) in 5 (0.4%); ixazomib, lenalidomide, and dexamethasone (IRD) in 4 (0.3%); and daratumumab, bortezomib, melphalan, and prednisone (DVMP) in 1 (0.1%) (Supplementary Table).

### Survival outcomes by percentage of CPCs

**Fig. 1:**
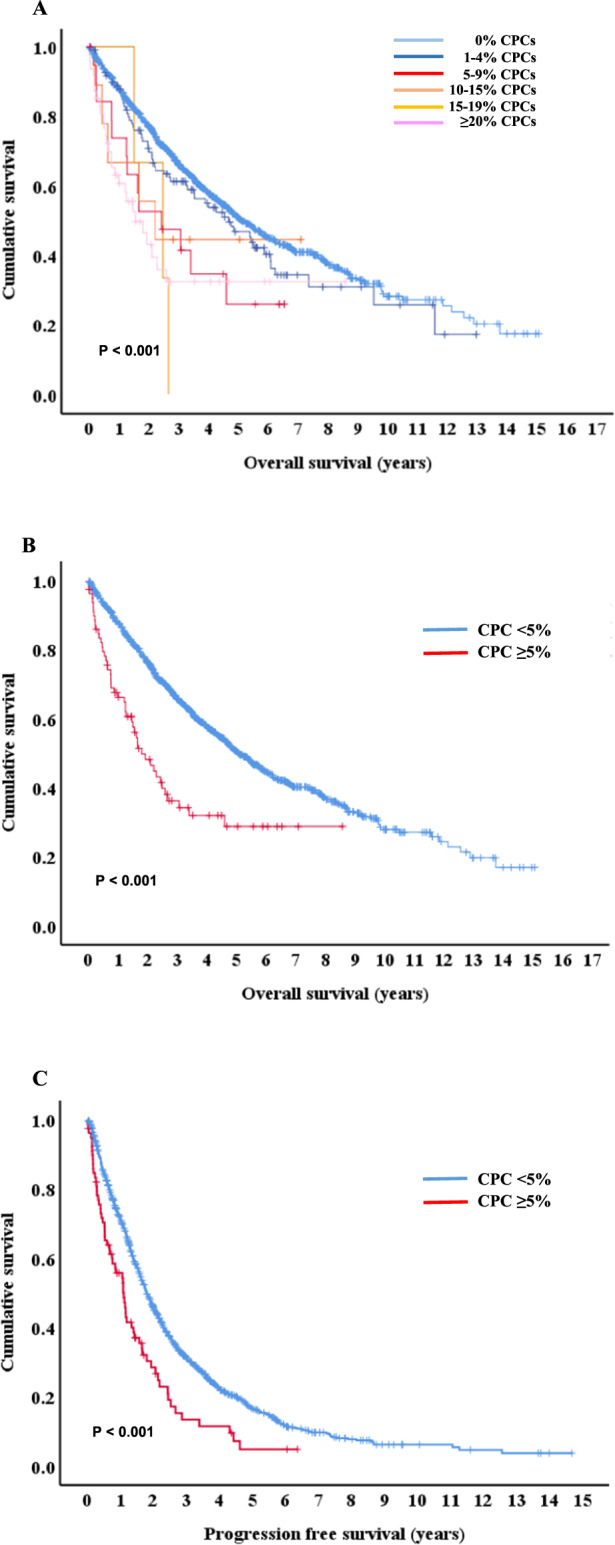
Survival outcomes by the percentage of circulating plasma cells. Kaplan-Meier survival curves for overall survival (OS) based on the percentage of circulating plasma cells (CPCs) (**A**), OS for patients with CPCs ≥ 5% (**B**), and progression free survival for patients with CPCs ≥ 5% (C).

Over a median follow-up of 48.3 months, the median PFS and OS were 21.0 months (19.6–22.4) and 58.6 months (52.9–64.3). The median OS for patients with 0%, 1–4%, 5–9%, 10–14%, 15–19%, and ≥ 20% CPCs were 61.2 (95%CI 55.0–67.4), 56.1 (95%CI 39.5–72.8), 29.2 (95%CI 5.5–52.9), 26.4 (95%CI 7.2–45.7), 29.7 (95%CI 10.9–48.4), and 18.5 (95%CI 8.7–28.3) months, respectively (Fig. 1A, *P* < 0.001). The OS was clearly divided by 5% of CPCs (60.9 months in patients with CPCs <5% vs. 21.5 months in patients with CPCs ≥ 5%, *P* < 0.001, Fig. 1B). In addition, patients with CPCs ≥ 5% had significantly lower median PFS compared to those with CPCs < 5% (13.1 months vs. 21.5 months, *P* < 0.001, Fig. 1C). The ED rate was significantly higher in patients with CPCs ≥ 5% than in those with CPCs <5% (13.9% vs. 4%, *P* = 0.001).

### Baseline clinical characteristics of patients with CPCs ≥ 5%

We evaluated the differences in baseline clinical and laboratory characteristics based on CPCs (Table 1). Median age was similar between the two groups. Light chain type was more frequent in patients with CPCs ≥ 5%, and 39.2% of patients with CPCs ≥ 5% had light chain only. Patients with CPCs ≥ 5% had significantly higher total calcium, serum creatinine, and lactate dehydrogenase (LDH) levels, and lower platelet counts than those with CPCs < 5%. Organomegaly was more common in patients with CPCs ≥ 5% (37.3% vs. 7.7%, *P* < 0.001). The presence of plasmacytoma at diagnosis was not significantly different (25.0% vs. 24.5%, *P* = 0.892), but the proportion of EMD was significantly higher in patients with CPCs ≥ 5% than in those with CPCs <5% (42.1% vs. 18.9%, *P* = 0.033). The sites of EMD in patients with CPCs ≥ 5% were the liver, soft tissue, stomach, lymph node, kidney, pleura, spleen and cranial nerve. R-ISS II and III, and high-risk cytogenetics, particularly del(17p) (25.0%) and t(11;14) (33.3%), were also more common among patients with CPCs ≥ 5%.

**Table 1:** Baseline clinical characteristics based on circulating plasma cells (CPCs) (*n* = 1,357).

Variables	0–5% (*n* = 1,278)	≥5% CPCs (*n* = 79)	*P* value
Median age, years (range)	64 (34–91)	63.5 (39-86)	0.946
Male, n (%)	718 (56.1)	45 (56.9)	0.908
Immunoglobulin (Ig) type, n (%)			0.003
IgG	723 (56.5)	29 (36.7)	
IgA	261 (20.4)	16 (20.2)	
IgM	8 (0.6)	1 (1.3)	
IgD	18 (1.4)	1 (1.3)	
Light chain only	260 (20.3)	31 (39.2)	
ECOG PS ≥ 2, n (%)	323 (25.3)	27 (34.6)	0.070
Plasmacytoma, n (%)	302 (24.5)	19 (25.0)	0.892
EMD	57 (18.9)	8 (42.1)	0.033
Organomegaly, n (%)	95 (7.7)	28 (37.3)	<0.001
Platelets, × 10^9^/L, median (range)	187 (20–1,005)	100 (8.1–370)	<0.001
Calcium, mg/dL, median (range)	9.1 (5.8–16.9)	9.7 (6.2–16.0)	<0.001
Creatinine, mg/dL, median (range)	1.05 (0.3–21.8)	1.31 (0.4–11.5)	0.002
LDH > (1 × ULN), n (%)	294 (23.5)	36 (47.3)	<0.001
R-ISS, n (%)			<0.001
I	223 (17.4)	3 (3.7)	
II	769 (60.1)	37 (46.8)	
III	207 (16.1)	31 (39.2)	
High risk cytogenetics, *n* (%)	212 (23.2)	17 (34.0)	0.080
del17p	103 (11.7)	14 (25.0)	0.010
t(4;14)	127 (15.3)	5 (10.8)	0.528
t(14;16)	23 (2.8)	2 (4.4)	0.375
t(11;14)	92 (17.2)	8 (33.3)	0.056
amp1q	290 (37.7)	10 (25.0)	0.130
First-line treatment, *n* (%)			0.557
Thalidomide-based regimen	410 (32.1)	20 (25.3)	
Bortezomib-based regimen	518 (40.5)	41 (51.9)	
Rd	82 (6.4)	3 (3.8)	
VTD	258 (20.2)	15 (19.0)	
IRD	4 (0.3)	0	
CMP	5 (0.4)	0	
DVMP	1 (0.1)	0	
Performance of ASCT	497 (38.9)	25 (31.6)	0.234

*n*, number, *ECOG* Eastern Cooperative Oncology Group, *PS*, performance status, *EMD* extramedullary plasmacytoma, *WBC* white blood cell, *LDH* lactate dehydrogenase, *ULN* upper limit of normal value, *R-ISS* Revised International Staging System, *Rd* lenalidomide and dexamethasone, *VTD* bortezomib, thalidomide, and dexamethasone, *IRD* ixazomib, lenalidomide, and dexamethasone, *CMP* carfilzomib, melphalan, and prednisone, *DVMP* daratumumab, bortezomib, melphalan, and prednisone, *ASCT* autologous stem cell transplantation

### Prognostic factors for OS in patients with CPCs ≥ 5%

We evaluated the predictors of OS in 79 patients with CPCs ≥ 5% (Table 2). In univariate analysis, nine variables were significantly associated with OS, including the presence of plasmacytoma, thrombocytopenia, increased LDH, hypercalcemia, elevated serum β2-microglobulin (>5.5 mg/L), hypodiploidy, and del(17p). In multivariate analysis, the presence of plasmacytoma (HR 3.990, 95%CI 1.328–11.986, *P* = 0.014) and elevated serum β2-microglobulin (HR 2.942, 95%CI 1.030–8.398, *P* = 0.044) were significant predictors of OS.

**Table 2:** Univariate and multivariate analyses of predictors of overall survival in patients with CPCs ≥ 5% (*n* = 79).

Variables	Univariate		Multivariate	
	HR (95% CI)	*P* value	HR (95% CI)	P-value
Age ≥70 years	1.038 (0.539-1.997)	0.912		
Males	1.052 (0.592-1.872)	0.862		
ECOG PS ≥ 2	1.511 (0.832-2.743)	0.185		
Organomegaly	1.026 (0.561-1.876)	0.933		
Plasmacytoma	2.853 (1.503-5.418)	0.001	3.990 (1.328-11.986)	0.014
Leukocytosis (≥ 11×10^9^/L)	1.318 (0.711-2.441)	0.389		
Hemoglobin < 8.0 g/dL	0.882 (0.473-1.645)	0.690		
Platelets < 130×10^9^/L	2.636 (1.304-5.326)	0.007	1.986 (0.622-6.337)	0.247
LDH > (1×ULN)	2.923 (1.614-5.294)	<0.001	1.255 (0.572-2.753)	0.572
Serum creatinine ≥ 2 mg/dL	1.173 (0.597-2.304)	0.644		
Serum calcium > 11.0 mg/dl	2.081 (1.053-4.110)	0.035	1.413 (0.486-4.107)	0.525
Serum albumin < 3.5 g/dL	1.248 (0.707-2.204)	0.444		
Serum β2-microglobulin > 5.5 mg/L	4.250 (2.012-8.976)	<0.001	2.942 (1.030-8.398)	0.044
Hypodiploidy	3.019 (1.481-6.155)	0.002	1.985 (0.545-7.231)	0.299
Hyperdiploidy	1.563 (0.706-3.459)	0.271		
del17p	2.353 (1.128-4.909)	0.023	1.231 (0.369-4.105)	0.735
t(4;14)	1.732 (0.590-5.084)	0.318		
t(14;16)	0.846 (0.113-6.316)	0.871		
t(11;14)	1.855 (0.580-5.926)	0.297		
amp1q	1.279 (0.529-3.093)	0.585		

*ECOG* Eastern Cooperative Oncology Group, *PS* performance status, *LDH* lactate dehydrogenase, *ULN* upper limit of normal value, *CR* complete response, *ASCT* autologous stem cell transplantation

**Fig. 2:**
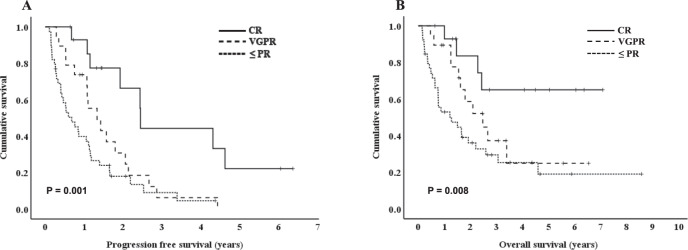
Survival outcomes by the response to induction therapy in primary plasma cell leukemia. Kaplan-Meier survival curves for progression-free survival (**A**) and overall survival (**B**) based on the response to induction therapy in patients with circulating plasma cells ≥ 5%.

The PFS and OS were significantly higher in patients achieving the deep response than those with less than a partial response (PR) [PFS; 29.3 months in CR vs. 15.9 months in very good partial response (VGPR) vs. 8.1 months in ≤ PR, *P* = 0.001, OS; not reached in CR, 29.7 months in VGPR vs. 15.2 months in ≤ PR, *P* = 0.008, Fig. 2A, B]. In the group aged < 65 years, the median OS for patients who underwent ASCT was significantly longer than those who did not receive ASCT (not reached vs. 9.2 months, *P* < 0.001).

**Fig. 3:**
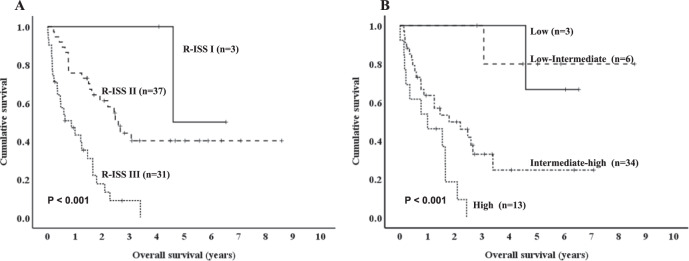
Prognostic impact of the Revised International Staging System (R-ISS) and Second Revision of the International Staging System (R2-ISS) in primary plasma cell leukemia. Kaplan-Meier survival curves for overall survival based on the Revised International Staging System (R-ISS) (**A**) and Second Revision of the International Staging System (R2-ISS) (**B**) in patients with circulating plasma cells ≥ 5%.

We also analyzed the prognostic impact of R-ISS and R2-ISS in patients with CPCs ≥ 5%. Although R-ISS I was uncommon, R-ISS was prognostic for OS in 71 patients with CPCs ≥ 5% (55.2 months in R-ISS I vs. 31.1 months in R-ISS II vs. 10.4 months in R-ISS III, *P* < 0.001, Fig. 3A). R2-ISS was also predictive of OS in 56 patients with CPCs ≥ 5% (Median OS, not reached in the low and low-intermediate groups vs. 21.5 months in the intermediate-high group vs. 12.0 months in the high group, *P* < 0.001, Fig. 3B).

## Discussion

The revised diagnostic criteria for PCL are based on the results of two retrospective studies. A Spanish study reviewed the clinical outcomes of 482 patients classified into four groups on the basis of CPCs in PBS: 0%, 1–4%, 5–20%, and ≥ 20% [5]. A study by the Mayo Clinic classified 176 patients into three groups: 1–4%, 5–19%, and ≥ 20% CPCs [6]. The two studies reported a similar median OS for the 5-20% and ≥ 20% groups, which were significantly inferior compared to those with CPCs <5%. A recent retrospective study verified the revised diagnostic criteria. In total, 158 patients (7.0%) had CPCs ≥ 5% and significantly lower PFS and OS than those with MM [11]. However, these studies included patients who received conventional chemotherapy as first-line therapy; the proportion was particularly high in the Mayo Clinic study (58%). In the present study, we evaluated the significance of the revised diagnostic criteria in patients who received induction therapy with immunomodulatory drugs (IMiDs) or proteasome inhibitors (PIs). In addition, patients with 5–20% CPCs were further classified into three groups: 5–9%, 10–14%, and 15–19%. The median OS for patients with CPCs ≥ 20% was 1.8 years, slightly longer than that reported in the two studies mentioned above (1.1 years and 1.4 years, respectively), but significantly lower compared to those with CPCs < 5%. This confirms that 5% is the optimal CPC cut-off value for PCL diagnosis in patients treated with IMiDs or PIs.

In general, different diseases may have clinical and genetic differences that it can be a clue for diagnosis or affect the diagnostic criteria. Previous studies showed that primary PCL occur in younger patients with unfavorable clinical characteristics [4, 12, 13]. However, the Spanish study did not show any differences in clinical characteristics, including age, sex, LDH level, and Durie-Salmon and ISS stages among the four groups. In the present study, patients with CPCs ≥ 5% had more advanced R-ISS stages frequently accompanied by thrombocytopenia, hypercalcemia, renal failure, elevated LDH levels and organomegaly. In addition, del(17p) and t(11;14) were more common in primary PCL diagnosed using the revised criteria than in MM. Recently, high-throughput genomic analyses, including transcriptomic studies, gene-expression profiling, and whole exon-sequencing, have been used to investigate the biological features of primary PCL [14–17]. Hofste et al. [18] performed transcriptomic analyses of newly diagnosed MM and primary PCL diagnosed using the previous criteria, and reported that some patients with newly diagnosed MM had PCL-like transcriptomic profiles. This may have a new prognostic value in addition to the existing prognostic factors in newly diagnosed MM and also may suggested new diagnostic criteria of PCL based on genetic differences.

We evaluated the prognostic impact of achievement of deep response in primary PCL patients diagnosed using the revised criteria. CR after induction therapy was associated with improved OS. Although the optimal induction therapy for primary PCL is controversial, IMiDs and PIs have demonstrated efficacy for the treatment of primary PCL [19, 20]. A combination of an IMiD and PI may be the optimal induction therapy for primary PCL. In the EMN12/HOVON129 study, the combination of carfilzomib, lenalidomide, and dexamethasone (KRD) showed remarkable efficacy, with an overall response rate of 93%, CR of 33% and VGPR of 55% [21]. In addition, ASCT after induction therapy and maintenance therapy reduced early relapse and improved survival in primary PCL patients [22–25]. Our study also showed the importance of ASCT, but did not evaluate the role of maintenance therapy because of the small, heterogeneous sample. Further prospective studies are required to determine the optimal treatment strategy for primary PCL diagnosed using the revised criteria.

R-ISS, based on ISS stage, cytogenetic abnormalities, and serum LDH levels at diagnosis, is a reliable prognostic system for predicting survival in patients with MM [8]. However, it has the limitation that a large number of heterogeneous patients are classified as R-ISS stage II. Therefore, R2-ISS was developed to improve risk stratification for newly diagnosed MM patients, particularly those at intermediate risk [9]. The present study evaluated the prognostic value of R-ISS and R2-ISS in primary PCL diagnosed using the revised criteria, and found that both were predictive of OS. Although a large number of patients were classified into the advanced stage, the prognostic systems for MM, including R-ISS or R2-ISS may be also helpful in predicting survival of primary PCL patients.

In addition to the retrospective design and small sample size, this study had some other limitations. Cytogenetic abnormalities could not be evaluated in all patients; therefore, we could not demonstrate any significant genetic differences between PCLs and MMs. Also, a small number of patients underwent ASCT, because only patients aged < 65 years were covered for ASCT by the insurance system. The role of tandem or allogeneic stem cell transplantation was also not evaluated. Previous studies have reported that tandem or allogeneic stem cell transplantation may be useful for the treatment of PCL [26]. However, there was no patients who underwent tandem or allogeneic stem cell transplantation in this study.

In conclusion, we evaluated the significance of the revised cut-off value for primary PCL diagnosis in 1,357 patients who received induction therapy with IMiDs or PIs. Among these patients, 187 (13.8%) had CPCs at diagnosis. Patients with CPCs ≥ 5% had significantly lower median PFS and OS than those with CPCs < 5%. This work provides evidence in support of the revised diagnostic criteria for primary PCLs.

## Supplementary information

Supplementary Table

## Data Availability

The data may be obtained from the corresponding authors on reasonable request.
