# Supplementary material for: Validation of the revised diagnostic criteria for primary plasma cell leukemia by the Korean Multiple Myeloma Working Party
Source: Blood Cancer J. 2022 Nov 21;12(11):157. doi: 10.1038/s41408-022-00755-w (PMC9676183; doi:10.1038/s41408-022-00755-w)
Supplement: Supplementary file 1 — Supplementary Table [file 41408_2022_755_MOESM1_ESM.docx]

Supplementary Table. Induction treatments in all patients.

| Induction therapy for all patients (n=1,357) | n (%) |
| --- | --- |
| Thalidomide, Cyclophosphamide, Dexamethasone  Thalidomide, Dexamethasone  Thalidomide, Melphalan, Prednisolone  Thalidomide, Doxorubicin, Dexamethasone  Bortezomib, Melphalan, Prednisolone  Bortezomib, Cyclophosphamide, Dexamethasone  Bortezomib, Dexamethasone  Bortezomib, Doxorubicin, Dexamethasone  Lenalidomide, Dexamethasone  Bortezomib, Thalidomide, Dexamethasone  Ixazomib, Lenaliomide, Dexamethasone  Carfilzomib, Melphalan, Prednisolone  Daratumumab, Bortezomib, Melphalan, Prednisolone | 290 (21.4)  119 (8.8)  18 (1.3)  3 (0.2)  512 (37.7)  31 (2.3)  13 (1.0)  3 (0.2)  85 (6.3)  273 (20.1)  4 (0.3)  5 (0.4)  1 (0.1) |
| Induction therapy in patients CPCs ≥ 5% (n=79) |  |
| Bortezomib, Melphalan, Prednisolone  Bortezomib, Thalidomide, Dexamethasone  Thalidomide, Cyclophosphamide, Dexamethasone  Thalidomide, Dexamethasone  Bortezomib, Cyclophosphamide, Dexamethasone  Thalidomide, Doxorubicin, Dexamethasone  Bortezomib, Dexamethasone  Lenalidomide, Dexamethasone  Bortezomib, Doxorubicin, Dexamethasone | 33 (41.8)  15 (19.0)  12 (15.2)  5 (6.3)  4 (5.1)  3 (3.8)  3 (3.8)  3 (3.8)  1 (1.3) |

Abbreviation: n, number.
